# Angiogenesis, Cancer, and Vascular Aging

**DOI:** 10.3389/fcvm.2017.00065

**Published:** 2017-10-24

**Authors:** Junji Moriya, Tohru Minamino

**Affiliations:** ^1^Office of Cellular and Tissue-Based Products, Pharmaceuticals and Medical Devices Agency, Tokyo, Japan; ^2^Department of Cardiovascular Biology and Medicine, Niigata University Graduate School of Medical and Dental Sciences, Niigata, Japan

**Keywords:** aging, therapeutic angiogenesis, cellular senescence, angiogenic factors, endothelial progenitor cells, cancer

## Abstract

Several lines of evidence have revealed that the angiogenic response to ischemic injury declines with age, which might account for the increased morbidity and mortality of cardiovascular disease (CVD) among the elderly. While impairment of angiogenesis with aging leads to delayed wound healing or exacerbation of atherosclerotic ischemic diseases, it also inhibits the progression of cancer. Age-related changes of angiogenesis have been considered to at least partly result from vascular aging or endothelial cell senescence. There is considerable evidence supporting the hypothesis that vascular cell senescence contributes to the pathogenesis of age-related CVD, suggesting that vascular aging could be an important therapeutic target. Since therapeutic angiogenesis is now regarded as a promising concept for patients with ischemic CVD, it has become even more important to understand the detailed molecular mechanisms underlying impairment of angiogenesis in older patients. To improve the usefulness of therapeutic angiogenesis, approaches are needed that can compensate for impaired angiogenic capacity in the elderly while not promoting the development or progression of malignancy. In this review, we briefly outline the mechanisms of angiogenesis and vascular aging, followed by a description of how vascular aging leads to impairment of angiogenesis. We also examine potential therapeutic approaches that could enhance angiogenesis and/or vascular function in the elderly, as well as discussing the possibility of anti-senescence therapy or reversal of endothelial cell senescence.

## Introduction

There is accumulating evidence that angiogenesis, which is the process of forming new blood vessels from existing vascular structures, declines significantly with aging (1–6). Aging is a major risk factor for various diseases. In the United States, people over 65 years old have a higher prevalence of cardiovascular disease (CVD), and the prevalence of CVD will increase by nearly 10% over the next two decades (7). Age-dependent impairment of angiogenesis is considered to be one of the main contributors to increased cardiovascular morbidity and mortality. Therefore, understanding the mechanisms by which aging induces pathophysiological changes of the vascular system, including impairment of angiogenesis, is critical for developing therapeutic strategies to manage age-related CVD. This review outlines the mechanisms of angiogenesis and vascular aging or endothelial cell senescence. Then recent evidence for the association between vascular aging and angiogenesis is described, followed by discussion about the potential to develop therapeutic angiogenesis and anti-senescence therapy for age-related CVD.

## Angiogenesis in the Elderly

### Major Mechanisms of Neovascularization

Growth of new blood vessels in response to certain stimuli such as tissue ischemia is called neovascularization and is categorized into three mechanisms, which are angiogenesis, arteriogenesis, and vasculogenesis (8). Angiogenesis occurs in small capillaries and involves sprouting of existing vascular endothelial cells (9). Arteriogenesis is a mechanism by which larger arteries form collateral vessels to maintain the blood supply after occlusion (10), while vasculogenesis involves the creation of vessels mainly by endothelial progenitor cells (EPCs), which were first reported in 1997 (11). The putative EPCs were initially typified by expression of CD34 and vascular endothelial growth factor receptor-2 (VEGFR-2) (11), then specified by additional various other markers expressed on their surface, such as CD 133, CD31, and von Willebrand factor (12, 13). It is known that EPCs arise from the bone marrow and differentiate into endothelial cells which form *de novo* vascular structures (8, 14). All three mechanisms are believed to contribute to the development of physiological or pathological neovascularization.

### Effects of Aging on Angiogenesis

Recovery of blood flow after hind limb ischemia is reported to be impaired in older animals compared with young animals (1). Consistent with this finding, the development of collateral arteries is significantly impaired in older patients with coronary artery disease (15), and the incidence of amputation is high in elderly patients with acute lower limb ischemia (16). These observations support the notion that impairment of angiogenesis occurs with aging. Indeed, endothelial cells from aged mice show a decreased capacity for both proliferation and migration (2, 3). Moreover, impairment of angiogenesis with aging contributes to delayed wound healing. Healing of skin wounds is a process that involves aggregation of keratinocytes and fibroblasts through the formation of highly vascular granulation tissue (2, 17, 18). Many aspects of the healing process are influenced by aging, and it was reported that formation of benign granulomas is inhibited in aged mice along with a decrease of capillary density (2).

On the other hand, progression of cancer is generally slower in the elderly compared with younger patients, although the incidence of cancer increases with age. In addition to the reduced capacity of tumor cells for proliferation and migration, impairment of angiogenesis is considered to have an important role in slowing the growth of cancer in elderly patients (19). Angiogenesis is essential for tumor progression, and there is an association between tumor vascularity and the prognosis of most neoplasms. In aged mice, invasion of malignancies is suppressed along with a decrease of tumor vasculature (19), and a reduced tumor microvessel count was reported in elderly patients with breast cancer (20). Thus, impairment of angiogenesis in the elderly is likely to have an influence on the prognosis of cancer, and attenuation of angiogenic capacity with aging can be seen as a mechanism that inhibits tumor progression.

## Molecular Mechanisms of Vascular Aging

The age-related reduction of angiogenic capacity and endothelial function is believed to at least partly stem from a phenomenon called vascular aging (21), which is characterized by cellular senescence affecting the vascular endothelium. In cultured cells, cellular senescence is the term for irreversible growth arrest that occurs after a certain number of cell division cycles (22). Senescent cells exhibit both morphological changes and phenotypic alterations associated with differences of gene expression (23). It is known that the lifespan of cultured cells is negatively correlated with the age of the donor, and that primary cultured cells from patients with premature aging syndromes have a significantly shorter lifespan (24, 25). These observations have led to the hypothesis that cellular senescence is associated with the aging processes, which was first postulated in the 1960s (26, 27). This hypothesis has been extensively investigated during the past few decades, leading to improved understanding of the molecular mechanisms underlying cellular senescence. The biological significance of cellular senescence is recognized to be its role as a protective mechanism against carcinogenesis due to DNA damage or various cellular stresses (28, 29). However, a recent study revealed that anti-inflammatory therapy with canakinumab could significantly reduce incident lung cancer in patients with atherosclerosis (30). Because aging is known to promote vascular inflammation by increasing reactive oxygen species (ROS) production (6), chronic inflammation during vascular aging might promote progression of cancer.

One of the most widely discussed hypotheses that could explain vascular cell senescence is the telomere hypothesis (31). Telomeres are chromatin complexes composed of non-nucleosomal DNA (TTAGGG repeats) and various telomere-binding proteins that are located at the ends of chromosomes and contribute to genomic stability by protecting this region from degradation and recombination (32). Telomeres become shorter with each cell division, possibly due to imperfect duplication of the extreme terminals of the chromosomes by DNA polymerase. Progressive telomere shortening eventually triggers senescence and reduces the proliferative capacity of cells (33). Telomerase is an enzyme that elongates telomeres by using its RNA component as a template (34). Introduction of telomerase into human endothelial cells inhibits telomere shortening with cell division and protect against senescence, suggesting that telomeres may have an important role in vascular cell senescence (35–37).

In addition to the telomere hypothesis, some telomere-independent mechanisms of vascular aging have been suggested. Angiotensin II induces premature senescence of human vascular smooth muscle cells without affecting telomere length by upregulating p53/p21 expression and activating nuclear factor kappa B to increase proinflammatory cytokine production (38). Senescence of human vascular endothelial cells was also reported to involve activation of Akt, suggesting that insulin/Akt signaling may be important in regulating the lifespan of these cells (39). This mechanism is reported to be related to control of the production of ROS (39).

## Mechanism of Impaired Angiogenesis Associated with Vascular Aging

Several mechanisms have been proposed as potential underlying causes of the age-related impairment of angiogenesis.

### Reduced Production/Response to Growth Factors and Nitric Oxide (NO)

It is known that expression of vascular growth factors and/or the response to these factors is attenuated in elderly persons. Production of vascular endothelial growth factor (VEGF), which is one of the key regulators of physiological and pathological angiogenesis (40–42), is decreased in the elderly at both basal levels and in response tissue injury (43–45). This is thought to be due to reduced activation of hypoxia-inducible factor-1α, a transcription factor for VEGF (46, 47). Also, expression of platelet-derived growth factor is inhibited in cardiac endothelial cells from aged rats (48), while the response of senescent human umbilical vein endothelial cells to basic fibroblast growth factor (FGF) is diminished due to impaired tyrosine phosphorylation of FGF receptors (49). All of these changes are likely to contribute to impairment of angiogenesis in the elderly.

Moreover, production of NO is decreased in the vascular cells of elderly persons or in senescent endothelial cells (50–52). Reduced bioavailability of NO with aging not only inhibits vasodilation through its innate effect, but also increases the sensitivity of endothelial cells to apoptotic stimuli, leading to disruption of endothelial function and angiogenic potential (53).

### Reduced Number/Function of EPCs

Endothelial progenitor cells are cells recruited from the bone marrow to sites of ischemia that promote neovascularization by undergoing differentiation into endothelial cells (11, 54). EPCs are currently utilized for therapeutic angiogenesis as a form of cell transplantation therapy (55–57). EPCs obtained from elderly persons show reduced survival, migration, and proliferation in culture, suggesting functional impairment due to cellular senescence (58). Interestingly, the number and function of EPCs are inversely correlated with various risk factors for atherosclerosis (59–61). Exhaustion of these cells not only leads to impaired angiogenesis but also attenuates the maintenance of vascular homeostasis, which might result in initiation of atherosclerosis (62). Indeed, EPCs from patients with coronary artery disease show reduced proliferation and migration, while EPC numbers are decreased in patients with advanced coronary artery stenosis (63–65). The decline of EPC numbers is considered to result from impairment of differentiation in the bone marrow with aging, as well as attenuated recruitment of these cells due to reduced VEGF production in peripheral tissues. These changes could be partially explained by age-related alterations of the stem cell niche, such as decreased tenascin-C expression in bone marrow (66).

### Changes of the Extracellular Matrix

Endothelial cell proliferation requires a scaffold for cells to migrate and space for cells to grow, created by degradation of the basement membrane around blood vessels. Correct organization of the extracellular matrix has a critical influence on this process (67, 68). Because production of extracellular proteins such as fibronectin and collagen is known to decrease with aging, this change has been suggested to make a contribution to impairment of angiogenesis (69, 70).

Matrix metalloproteinases (MMPs) are proteases involved in degradation of the extracellular matrix (71). MMPs can be divided into several groups on the basis of cellular localization, biochemical properties, and sequence similarities (Table 1) (71). As well as production of extracellular proteins, the activity of MMPs decreases with aging (72). Conversely, the expression of tissue inhibitor of metalloproteinase, which inhibits MMPs, is enhanced by aging (73). The resulting dysregulation of MMPs is considered to be one of the key factors leading to impairment of angiogenesis in elderly persons, along with increased production of angiogenic inhibitors such as thrombospondins (3, 74, 75).

**Table 1:** Matrix metalloproteinase (MMP) family.

Enzyme	MMP
**I. Collagenase group**	
Interstitial collagenase (collagenase-1)	MMP-1
Neutrophil collagenase (collagenase-2)	MMP-8
Collagenase-3	MMP-13
**II. Gelatinase group**	
Gelatinase A	MMP-2
Gelatinase B	MMP-9
**III. Stromelysins**	
Stromelysin-1	MMP-3
Stromelysin-2	MMP-10
Stromelysin-3	MMP-11
**IV. Membrane-type (MT) MMPs**	
MT-1 MMP	MMP-14
MT-2 MMP	MMP-15
MT-3 MMP	MMP-16
MT-4 MMP	MMP-17
MT-5 MMP	MMP-24
MT-6 MMP	MMP-25
**V. Others**	
Matrilysin	MMP-7
Macrophage elastase (metalloelastase)	MMP-12
Enamelysin	MMP-20
Other human metalloproteases	MMP-18, MMP-19, MMP-23

### Cellular Senescence

Cellular senescence is believed to result from telomere shortening associated with successive cell division and chronic oxidative stress (76). Several studies have demonstrated that atherosclerotic lesions contain senescent vascular endothelial cells (77–79), and the telomere length of somatic cells is inversely correlated with the number of risk factors for atherosclerosis (80–86). In addition to the decline of replicative capacity, cellular senescence leads to increased expression of inflammatory cytokines and decreased production of NO by the vascular endothelium (87, 88). These changes associated with aging are considered to play a key role in the development of atherosclerosis, as well as directly leading to impairment of angiogenesis (22). EPCs also develop the functional and phenotypic characteristics of cellular senescence in elderly persons, resulting in impaired functioning of these cells (21, 58, 89).

## Therapeutic Implications of Age-Related Impairment of Angiogenesis

Atherosclerotic ischemic diseases, such as arteriosclerosis obliterans and ischemic heart disease, are among the major age-related diseases with surging morbidity and mortality (90). Atherosclerotic plaques from older patients tend to be larger with significant stenosis, as well as having more calcified lesions (91). Angiogenesis is promptly triggered by ischemia, but this response is attenuated in the elderly (1, 2, 5). While revascularization is currently the most effective treatment for ischemia, many patients are unsuitable for this therapy due to technical reasons or unclear benefit, especially among the elderly population (92). Wound healing is also impaired with aging, and this change is associated with reduced levels of angiogenic factors such as VEGF or FGF (2, 4).

Previous preclinical studies and small-scale clinical trials have shown that gene therapy or the delivery of VEGF or FGF protein, as well as cell therapy employing EPCs and bone marrow or peripheral blood mononuclear cells, have some efficacy for alleviating ischemia. These revascularization strategies are collectively called therapeutic angiogenesis (93–96). Moreover, local application of basic FGF to refractory skin ulcers has been shown to promote wound healing and has demonstrated remarkable clinical benefit (97–99), leading to approval of basic FGF as a topical treatment in Japan.

Unfortunately, therapeutic angiogenesis is not always effective. Among patients with critical limb ischemia, nearly half of those treated do not achieve sufficient improvement of ischemic symptoms (100). The key reasons for lack of improvement are considered to be an attenuated response to growth factors and decreased viability or function of transplanted cells due to cellular senescence (5). One of the potential strategies to overcome these problems is modification of senescence-associated molecules. Indeed, it has been reported that transduction of the human telomerase reverse transcriptase (TERT) gene into EPCs led to improvement of neovascularization in a murine model of hind limb ischemia (101), with this explicit anti-senescence strategy serving as a model for therapeutic angiogenesis.

**Figure 1:**
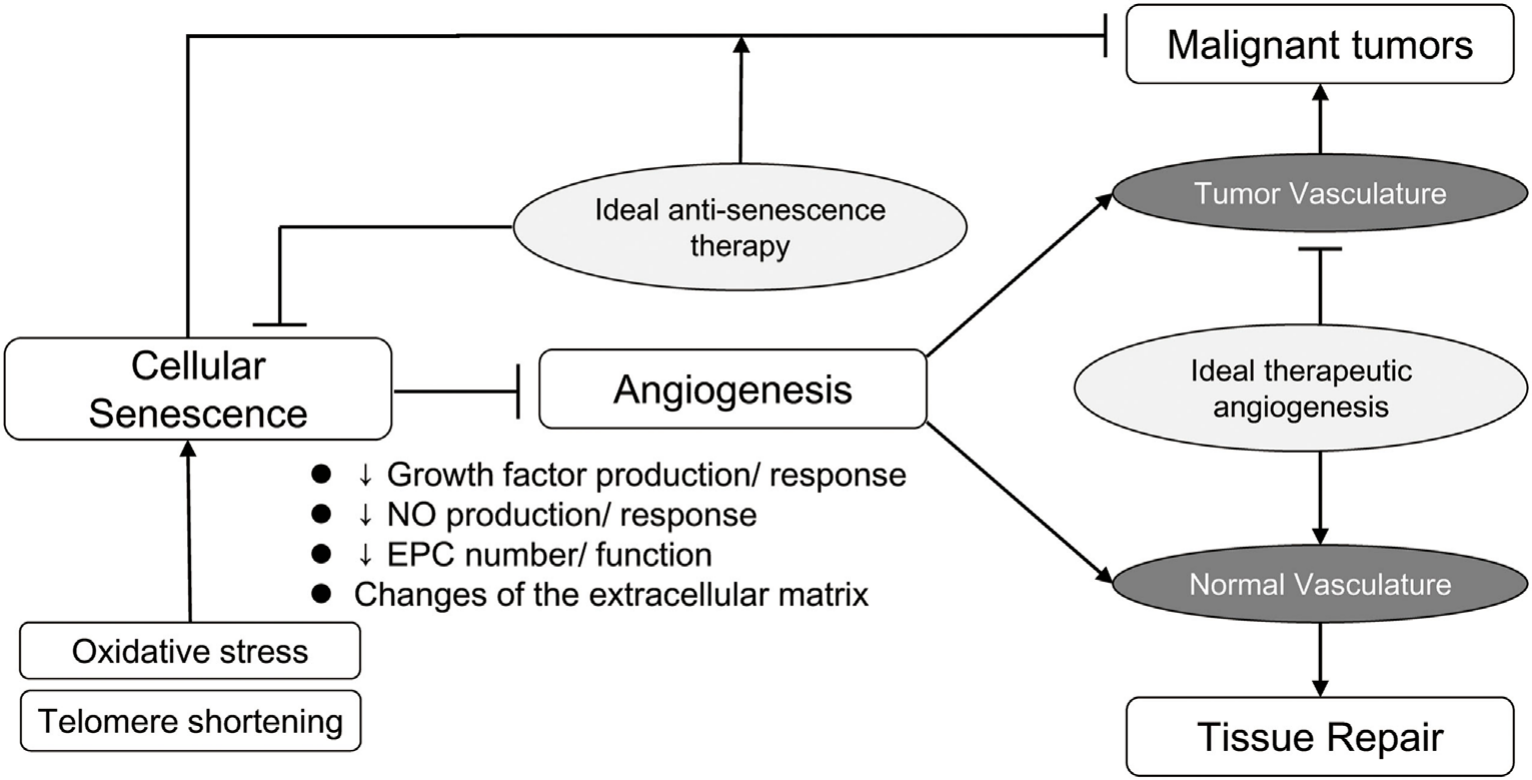
Ideal antisenescence therapy and therapeutic angiogenesis. Although cellular senescence and impaired angiogenesis have undesirable effects, these age-related changes also inhibit the progression of cancer. Restoring the repair potential of normal tissues, while preserving the protective effect against development and progression of malignant tumors, is the ultimate objective of anti-senescence therapy and therapeutic angiogenesis.

However, it should be noted that both cellular senescence and impairment of angiogenesis are mechanisms inhibiting cancer progression (4, 23). Strategies such as introduction of the TERT gene, as mentioned above, are thought to be associated with a high risk of cancer (101), and clinical application of this technology would be difficult in its present form. Thus, employing therapeutic angiogenesis in elderly patients will always be associated with a certain risk of promoting the development of cancer. Because therapeutic angiogenesis or anti-senescence therapy for the elderly is a two-edged sword, it is important to focus on therapeutic targets that are as specific as possible (Figure 1). Accordingly, local administration of these therapies could be one option. Additionally, various drugs with known cardioprotective effects, such as statins (102), thiazolidinediones (103), aspirins (104), and estrogens (105), have also been reported to increase telomerase activity and are not considered to increase the risk of malignancy. Thus, targeting the appropriate senescence-associated molecules may allow development of safe and effective anti-senescence therapy.

## Conclusion

Although impairment of angiogenesis with aging is detrimental for various ischemic diseases, it conversely has a favorable effect by suppressing the development and progression of malignant tumors. Deeper understanding of the detailed mechanisms involved in vascular aging and angiogenesis may lead to ideal molecular-targeted therapy that promotes angiogenesis by suppressing age-related signaling pathways while preserving the protective effect against cancer.

## Author Contributions

JM wrote the manuscript and TM revised the manuscript.

## Conflict of Interest Statement

The authors declare that the research was conducted in the absence of any commercial or financial relationships that could be construed as a potential conflict of interest.
